# Intelligent microbial cell factory with genetic pH shooting (GPS) for cell self-responsive base/acid regulation

**DOI:** 10.1186/s12934-020-01457-3

**Published:** 2020-11-02

**Authors:** Chenyi Li, Xiaopeng Gao, Xiao Peng, Jinlin Li, Wenxin Bai, Jiadong Zhong, Mengchao He, Ke Xu, Ying Wang, Chun Li

**Affiliations:** 1 Institute of Biochemical Engineering, School of Chemistry and Chemical Engineering, Beijing Institute of Technology, Beijing, 100081 People’s Republic of China; 2 School of Life Science, Yan’an University, Shanxi, 716000 People’s Republic of China; 3 Key Lab for Industrial Biocatalysis, Ministry of Education, Department of Chemical Engineering, Tsinghua University, Beijing, 100084 People’s Republic of China

**Keywords:** Genetic pH regulation, Base-regulating circuit, Acid-regulating circuit, Microbial cell factory, *Escherichia coli*

## Abstract

**Background:**

In industrial fermentation, pH fluctuation resulted from microbial metabolism influences the strain performance and the final production. The common way to control pH is adding acid or alkali after probe detection, which is not a fine-tuned method and often leads to increased costs and complex downstream processing. Here, we constructed an intelligent pH-sensing and controlling genetic circuits called “Genetic pH Shooting (GPS)” to realize microbial self-regulation of pH.

**Results:**

In order to achieve the self-regulation of pH, GPS circuits consisting of pH-sensing promoters and acid-/alkali-producing genes were designed and constructed. Designed pH-sensing promoters in the GPS can respond to high or low pHs and generate acidic or alkaline substances, achieving endogenously self-responsive pH adjustments. Base shooting circuit (BSC) and acid shooting circuit (ASC) were constructed and enabled better cell growth under alkaline or acidic conditions, respectively. Furthermore, the genetic circuits including GPS, BSC and ASC were applied to lycopene production with a higher yield without an artificial pH regulation compared with the control under pH values ranging from 5.0 to 9.0. In scale-up fermentations, the lycopene titer in the engineered strain harboring GPS was increased by 137.3% and ammonia usage decreased by 35.6%.

**Conclusions:**

The pH self-regulation achieved through the GPS circuits is helpful to construct intelligent microbial cell factories and reduce the production costs, which would be much useful in industrial applications.

## Background

pH control in the fermentation process is a widespread concern as its fluctuation results in numerous problems. Cell viability and production can be influenced by the surrounding pH. The activities of enzymes will decrease under an improper pH, and eventually, their biosynthetic efficiencies will reduce. Furthermore, an inappropriate pH can change the fermentation profile of microorganisms. For example, *Aspergillus Niger* can produce citric acid at an acidic pH, but a higher initial pH will lead to the accumulation of oxalic acid [1].

Therefore, a suitable environmental pH is essential for high fermentation productivity, and the optimization of pH in the fermentation process is critical. However, problems concerning pH fluctuations in fermentation are complex. During the regulation process, pH changes are nonlinearity along with the high sensitivity around neutral pH, leading to big challenges. To maintain a proper pH in fermentation processes, the most common method is the probe detection technique, which is combined with computer programs to directly add sterile acids and bases into fermenters to adjust the pH during fermentation [2]. However, this method mostly depends on electrode detection of pH, which requires a sufficient reaction time and can be easily interrupted by the environment, leading to the excessive addition of acid or base to the fermenter and thus affecting cell production. Moreover, the regulation process is usually continuously conducted until the pH reaches the set point [3]. This long-lasting fluctuation of pH, even over a small range, will require an unnecessarily excessive amount of acid and base. Moreover, the added acid or alkali will be neutralized and increase salt concentration in the system, resulting in a raise of osmotic pressures in cell cultivation and difficulties in downstream processing. Meanwhile, all of these methods mainly rely on electrical technology and increase costs and energy consumption. Therefore, a highly efficient and low-cost pH control method is needed.

One of the economic ways for pH control is microbial self-regulation to avoid the addition of acid or alkali solutions. The past decade has witnessed a blooming and rapid development of synthetic biology [4, 5]. New technologies in this field provide effective ways to both understand and deal with these challenges [6–8], such as the improvement of the production of some pharmaceutically important molecules and biofuels [9–12] and the development of functional genetic circuits [13–16]. Recently, researchers have developed a riboswitch-based method and generated a series of pH-sensing genetic devices for resisting acidic conditions [17]. Therefore, it is possible to develop pH self-regulation circuits using synthetic biology techniques. However, to our knowledge, there is still no reported pH self-regulation system up to now.

Herein, we designed an innovative and general strategy for bio-pH regulation. In detail, a self-regulating system, namely Genetic pH Shooting (GPS), was designed and constructed in *Escherichia coli,* which can sense pH change and promptly synthesize acidic or alkaline substances endogenously to adjust the improper pH. pH-responsive promoters and acid-/alkali-producing genes were two important parts of the genetic circuits. For the promoters responding to low or high pH environment, inducible promoters, P-asr and P-atp2, were employed. P-asr, a pH-responsive promoter native to *E. coli* str. K-12, induces gene transcription in acidic environments with a pH ranging from 5.0 to 6.0 [18, 19] and has low activity at neutral pH. The P-atp2 is an alkali-induced promoter, which has been proven to respond to pH ranging from 8.0 to10.0 in previous studies [20, 21]. These two pH responsive promoters were combined with the acid- or base- production genes to form the GPS system. The constructed GPS system was further employed in the production of lycopene, demonstrating its ability in enhancing the biosynthetic efficiency. The incorporation of GPS for pH control makes the host strain more robust, which is meaningful for better cell growth and enzyme activity. Moreover, our approach for pH control is subtle and can minimize the problems such as fluctuations resulted from excess addition of acid or alkali in traditional method. The GPS system could decrease the usage of extra acid and alkaline solutions in scale-up fermentation and thus will be helpful for simplifying the downstream processing. Therefore, intelligent microbial cell factories can be established employing this GPS system.

## Results

### Exploration and evolution of acid- and base-sensing promoters for cell self-responding pH

**Fig. 1:**
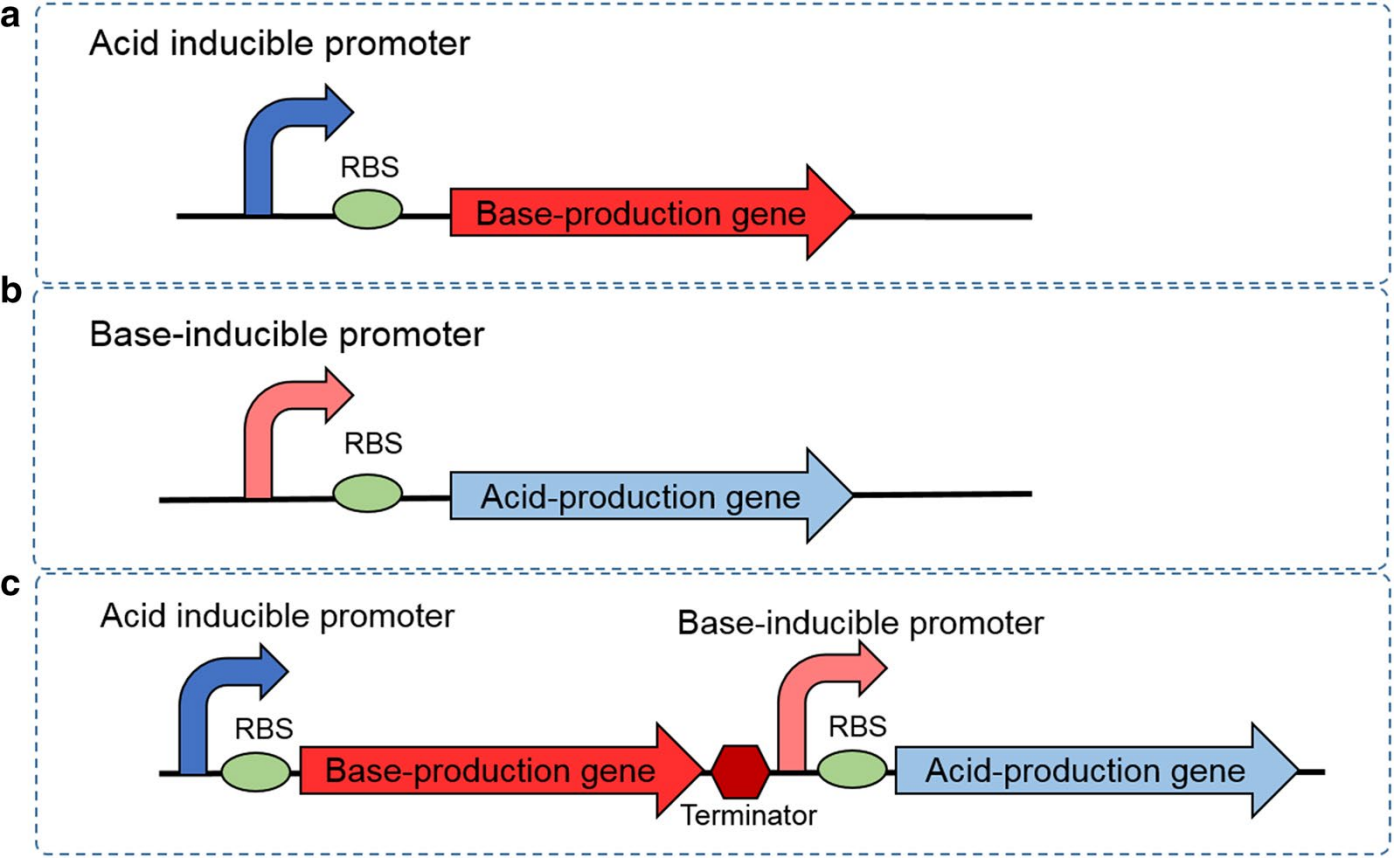
The intelligent pH-sensing and controlling genetic circuits. **a**: ASC, **b**: BSC, **c**: GPS

In the process of most microbial fermentations, pH regulation is important for high production and the common method of adding extra acid or alkali directly has many shortages. To achieve the self-regulation of pH during the cell fermentation process, a GPS circuit was designed in this study, including pH-sensing promoters that could respond to pH signals and produce acidic or alkaline substances to neutralize extra H^+^ or OH^−^ in cells (Fig. 1).

Inducible promoters are usually used in the regulation of gene expression under the induction of different signals. For example, P-asr induces gene transcription in acidic environments with a pH ranging from 5.0 to 6.0 [18, 19]. When the environmental pH is at a low level, the RstB, a histidine kinase protein, spontaneously senses the high proton concentration and is phosphorylated to activate RstA, the DNA-binding transcriptional regulator protein, which subsequently activate the function of P-asr promoter, driving the transcription of downstream genes [19]. P-atp2, an alkali-induced promoter from *Corynebacterium glutamicum* located in the F_0_F_1_ ATPase operon, responds to pH ranging from 8.0 to 10.0, particularly pH 9.0 [20, 21]. Hence, the pH-sensitive promoters (P-asr, P-atp2) were employed as biosensors to biologically sense the pH and control the functioning pH range, which could be incorporated into the GPS circuit.

**Fig. 2:**
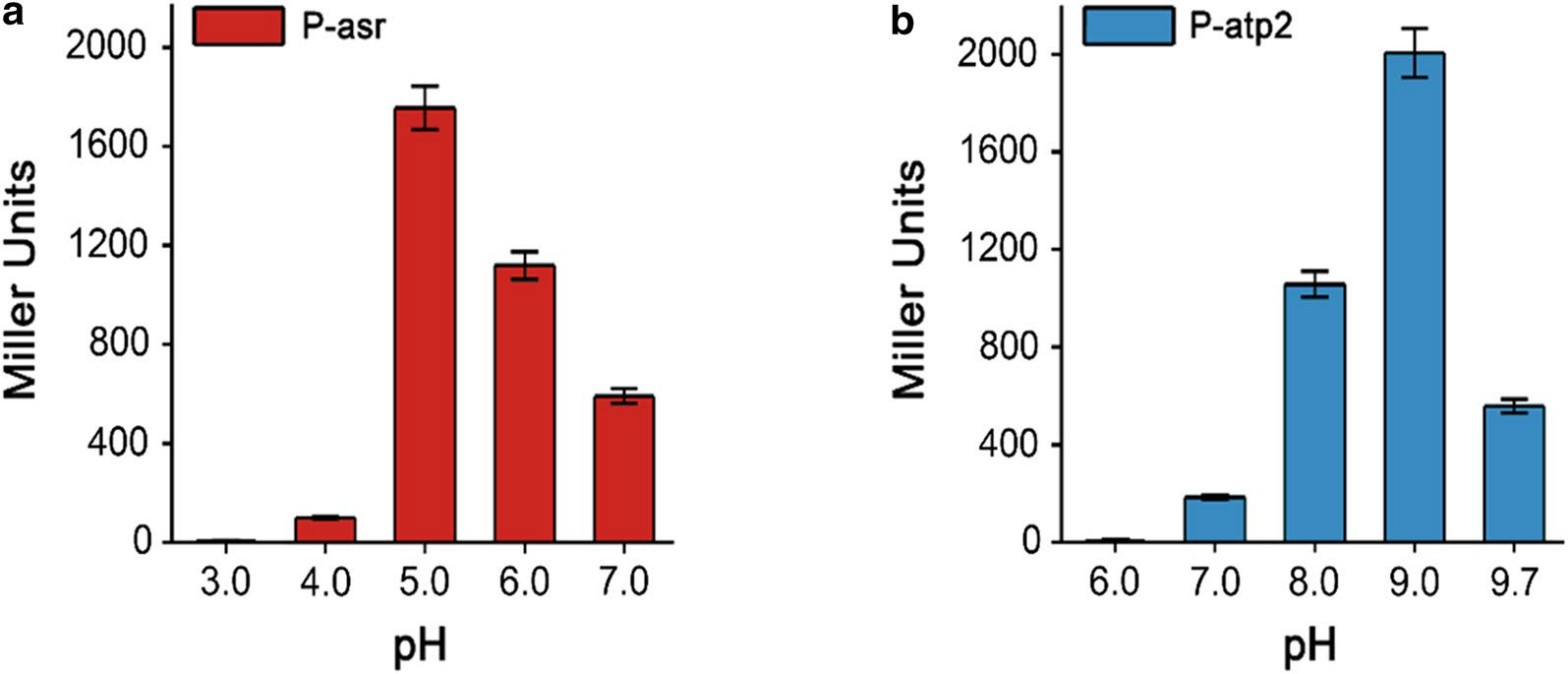
Relative enzyme activities at different pH values of acid-inducible promoter P-asr (**a**) and base-inducible promoter P-atp2 (**b**). The data represent the mean of three biological replicates

In order to precisely determine the inductive pH range and validate the efficiencies of these promoters at different pH values, the pH-sensing promoters were linked to the reporter gene *lacZα*, and the activities of the promoters at different pH values were verified by measuring the β-galactosidase activity. The results showed that P-asr had almost no activity at pH 3.0 and 4.0, while at pH 5.0, its highest activity was observed (Fig. 2a). The activity of P-asr gradually decreased with increasing pH. On the other hand, the activity of P-atp2, which can respond to high pH value (Fig. 2b), peaked at pH 9.0 and was relatively weaker at other pH values. Since most of the strains like *E. coli* seldom survive at pH 10.0, pH 9.7 was set as the highest pH value in this study.

**Fig. 3:**
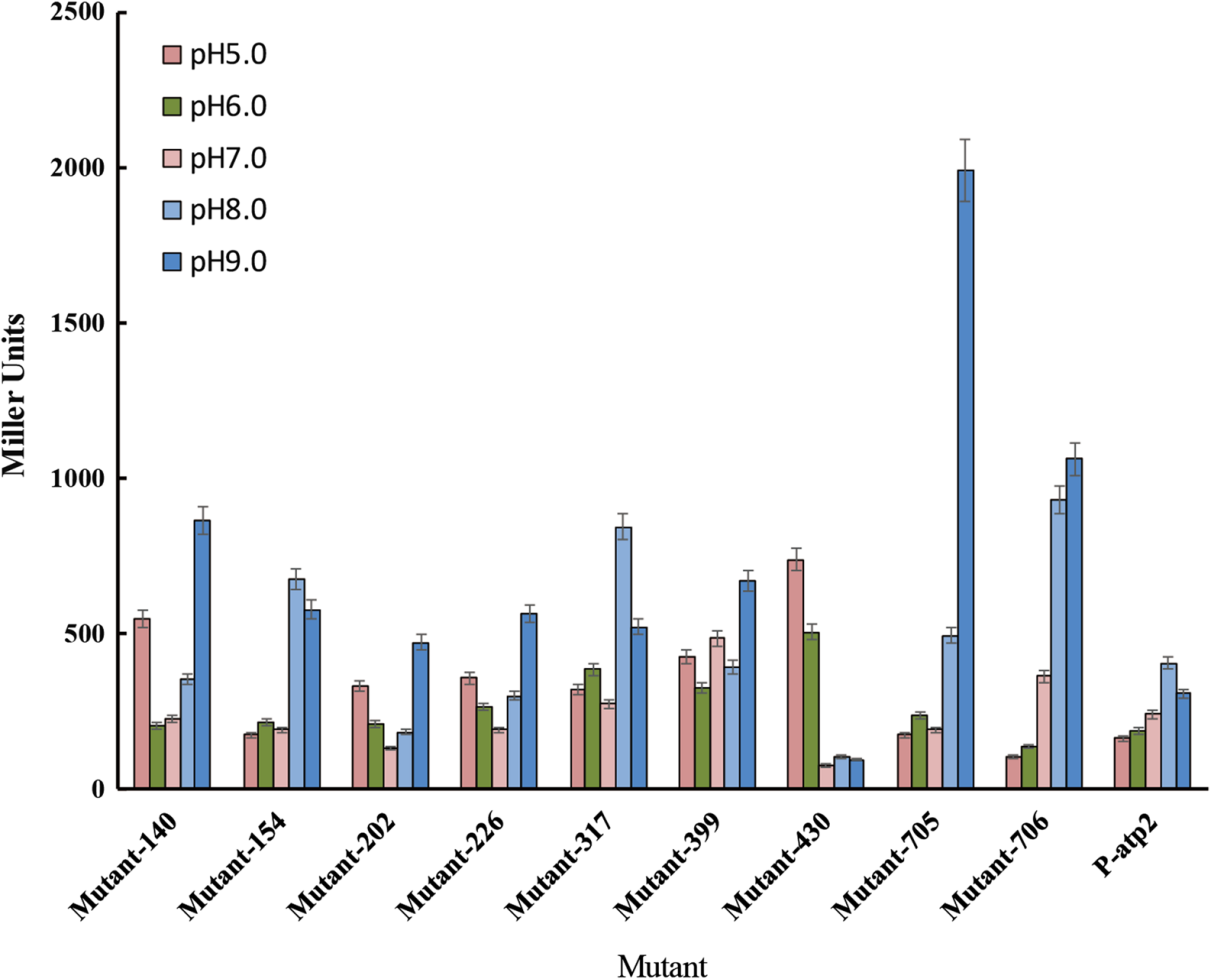
The screening for P-atp2 mutants under different pH values. These mutants were generated through error-prone PCR and selected through promoter activity assay. The data represent the mean of three biological replicates

Furthermore, a library of pH sensing promoters responding to diverse pH values was established through error-prone PCR, with the expectation of increasing the strict pH regulation capability of GPS (Fig. 3). Wild-type base-induced promoter P-atp2 was selected as the template for error-prone PCR, resulting in various promoters that had different strengths at different pH values (Fig. 3). Among the mutated promoters, mutant-430 had a more restricted pH responsive range than wild-type P-atp2. The highest promoter activity of mutant-430 only occurred at pH 9.0, and its response to environmental pH is not as sensitive as P-atp2. However, in our designed circuit, the wild-type P-atp2 is more suitable for our needs as we require the circuit’s quickly response to pH fluctuation during the fermentation. The P-atp2 can open the transcription of downstream genes when the pH reaches 8.0 and reaches highest activity at pH 9.0, while the mutant-430 can only be activated when pH reaches 9.0. Therefore, the original promoter P-atp2 was used for further study.

### Construction and characterization of base shooting circuit (BSC)

**Fig. 4:**
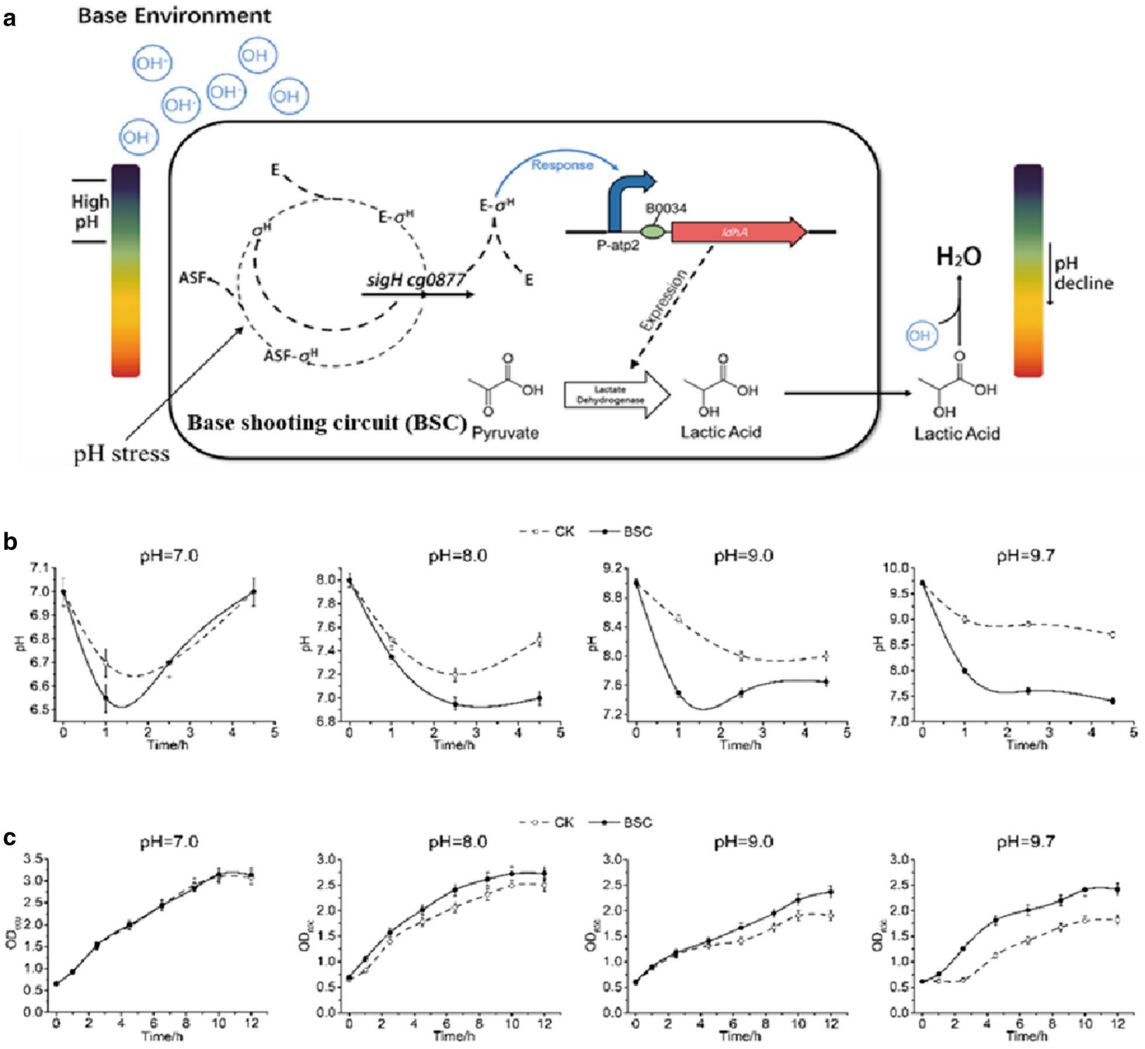
Effect of base-regulation circuit (BSC) on sensing and regulating base conditions. **a** Work principle of BSC. E represents the sigma factor sigE and ASF represents anti-sigma factor. **b** pH fluctuation curves at different initial pH values during the cultivation of *E. coli* harboring the BSC and the control check (CK) *E. coli* without BSC. **c** Growth curves for *E. coli* with and without BSC. The data represent the mean of three biological replicates

The base shooting circuit (BSC) responding to overly basic environment was designed and constructed to regulate the pH when it became higher than the optimal value (Fig. 1b). Two functional parts consisting of pH-responsive promoter sensing and responding to the basic environmental, i.e. high pH value, and the acid-producing gene regulating the improper pH by generating acids were included in the BSC. As described above, the P**-**atp2 promoter responding to high pH value was employed as alkali-responsive promoter in the BSC. In order to provide acidic compounds in base-shooting circuit (BSC), the lactate dehydrogenase gene *ldhA* was employed as the functional gene, forming the BSC (Fig. 4a).

Recombinant plasmid pUC-BSC was constructed by inserting the BSC into vector pSB1C3, and was then transformed into *E. coli* BM Top 10, resulting in recombinant *E. coli* BM-pSBBSC. The regulation functions of the BSC were characterized via cell growth and pH changes. *E. coli* BM-pSBBSC was cultivated under different alkaline pH conditions, respectively. A higher OD_600_ and relatively more stable pH were observed in recombinant *E. coli* BM-pSBBSC compared with the control strains at pH 8.0, 9.0 and 9.7, respectively. The results showed that pH was well-regulated after introducing BSC, which contributed to the cell growth (Fig. 4b, c). Hence, the constructed BSC worked well in *E. coli* and could facilitate cell growth. Moreover, the higher of the environmental pH, the functions of BSC were more significant (Fig. 4b, c). On the other hand, no obvious differences were observed in the growth curves between the engineered strain and the control ones at pH 7.0, which indicated that the BSC would not affect cell growth at neutral pH.

### Construction and characterization of acid shooting circuit (ASC)

**Fig. 5:**
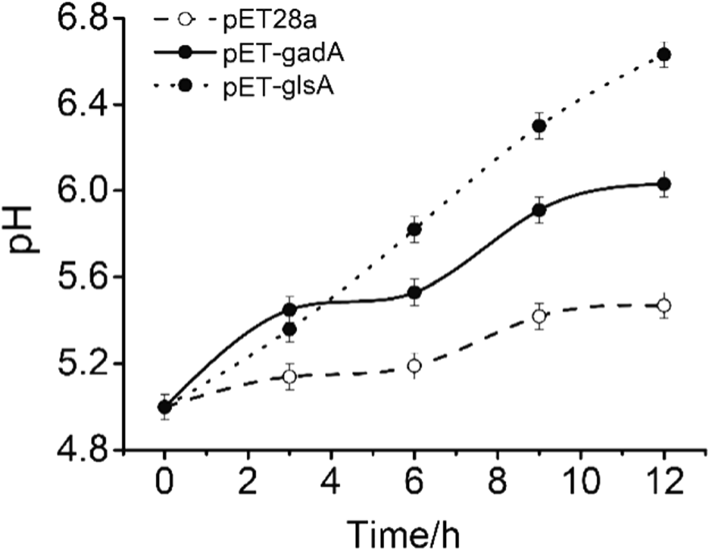
Acid regulation ability of *glsA* and *gadA.* A comparison of acid regulation ability between *glsA* and *gadA* with strains carrying empty plasmids as control. The data represent the mean of three biological replicates

**Fig. 6:**
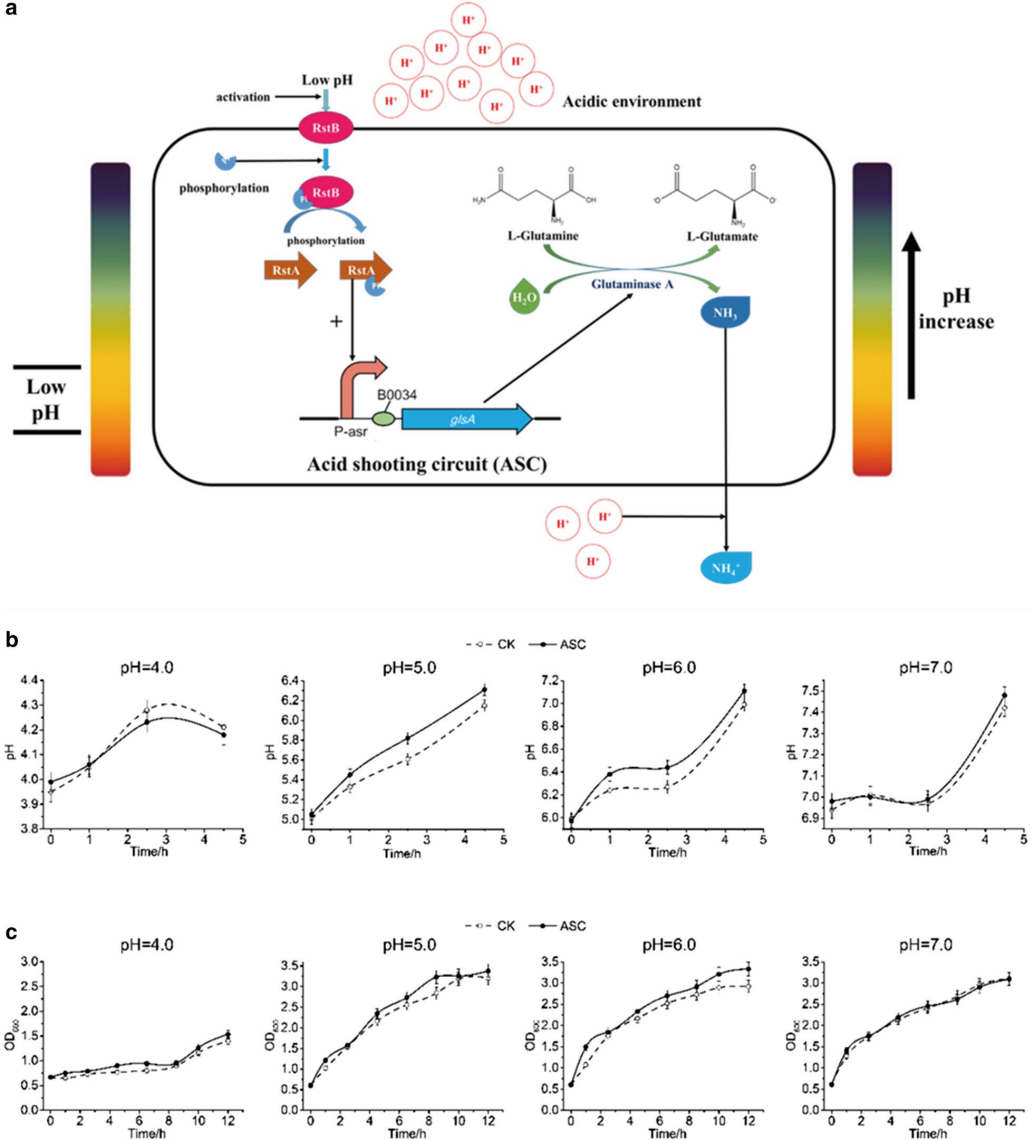
Effect of acid-shooting circuit (ASC) on sensing and regulating acidic conditions. **a** Work principle of ASC. **b** pH fluctuation curves at different initial pH values during the cultivation of *E. coli* harboring the ASC and the CK. **c** Growth curves for *E. coli* with and without ASC. The data represent the mean of three biological replicates

Similar to the BSC, acid shooting circuit (ASC) responding to overly acidic environment was also designed and constructed (Fig. 1a). P-asr responding to acidic environment served as acid-responsive promoter for ASC and the functional genes resisting acidic environment were mined, which were mainly base-producing genes. Two genes were selected: *glsA*, encoding glutaminase A, and *gadA*, encoding a decarboxylase that converts L-glutamate (Glu) to γ-aminobutyric acid (GABA), which is a zwitterionic molecule that reacts with acids. The two genes were cloned into the expression vector pET28a under T7 promoter and transformed into *E. coli* BL21(DE3), resulting in engineered BL-pETglsA and BL-pETgadA. The pH regulation ability of the two engineered strains was verified under the induction of 1 mM isopropyl β-D-1-thiogalactopyranoside (IPTG) at pH 5.0. *E. coli* BL21(DE3) harboring the empty vector was employed as the control strain. The results showed that the *glsA* gene had a relatively stronger ability to regulate the acidic environmental pH (Fig. 5). Thus, *glsA* was selected as the functional base producing gene in the ASC. The ASC was then constructed by ligating the acid-responding promoter P-asr and *glsA*, and transformed into *E. coli* BM Top10, generating strain BM-pSBASC (Fig. 6a).

The efficiency of ASC in regulating pH and cell growth was measured under different pH conditions (Fig. 6b, c). At pH 5.0 and 6.0, the engineered strain had a fast and strong ability to regulate the extracellular pH, implying that the ASC functions better at this pH range. There were little differences in both cell growth and pH fluctuations between the engineered strain and the control one at pH 4.0 and pH 7.0. As indicated in Fig. 2, the acid-inducible promoter P-asr had low activity at these pHs, which could be improved by mutations or replacing with the low pH-responsive promoters for the different pH ranges.

### Construction, characterization and application of genetic pH shooting (GPS) in Lycopene biosynthesis

**Fig. 7:**
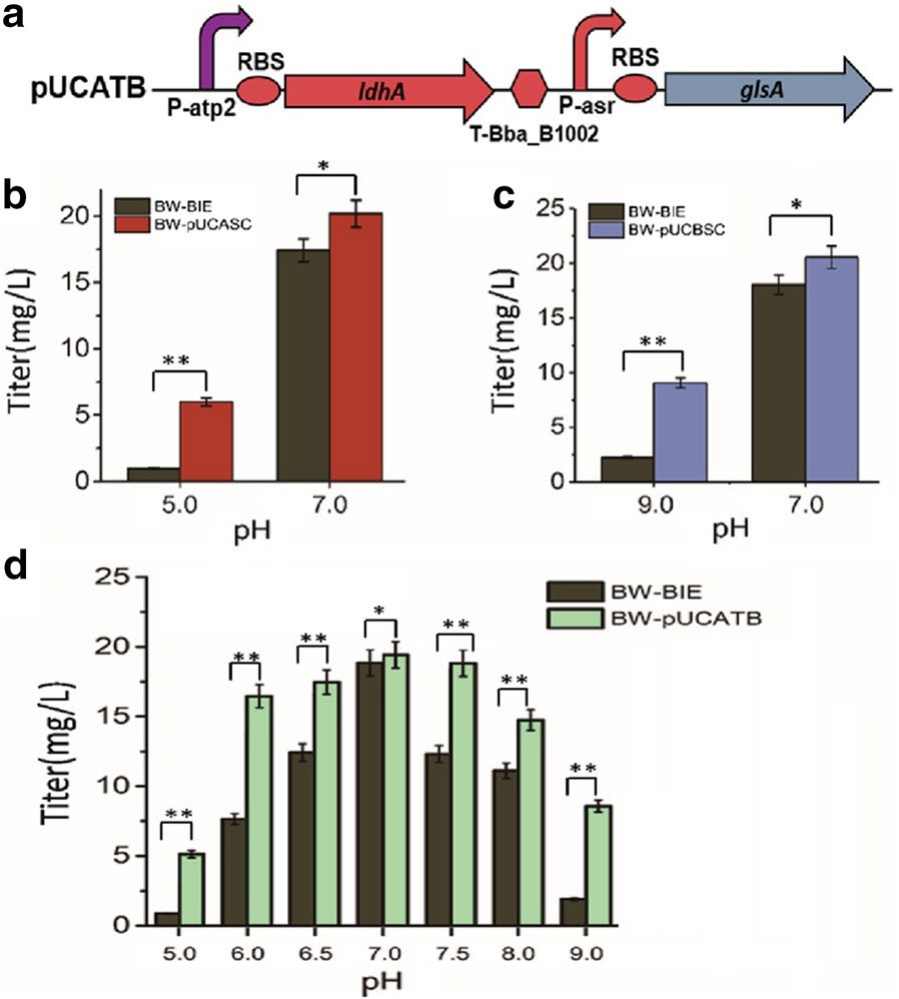
Application of GPS circuit on the lycopene production strain. **a** GPS circuit designed in this study. **b** Effect of ASC on lycopene production in acidic and neutral conditions. **c** Effect of BSC on lycopene production in alkali and neutral conditions. **d** Lycopene titers of BW-BIE and BW-pUCATB at different initial pH values. The data represent the mean of three biological replicates

During the fermentation processes, pH fluctuations often happen and can result in decreased productivities. Hence, general regulation circuits to achieve flexible pH regulation are needed. As described above, the pH-sensing promoters P-asr and P-atp2 can automatically respond to the environmental pH and induce the expression of *ldhA* and *glsA*, respectively, forming BSC or ASC. Based on the two circuits, GPS was designed and constructed combining the functions of BSC and ASC (Fig. 1c), resulting in recombinant plasmid pUCATB (Fig. 7a). For the construction of pUCATB, the strong terminator BBa_B1002 from the iGEM Registry was inserted into the middle noncoding area between ASC and BSC (Fig. 7a).

GPS could be employed to automatically maintain high biosynthetic efficiency under various non-optimal pH conditions while extensively decreasing the cost of artificial pH control. In order to test the applications of ASC, BSC and GPS in microbial cell factories for lycopene production, the single pH shooting circuit, ASC and BSC discussed above, were separately reconstructed on the plasmid pUC19 and then transformed into the *E. coli* BW-BIE, resulting in the recombinant strains *E. coli* BW-pUCASC and *E. coli* BW-pUCBSC. We tested the efficiency of the single pH shooting circuit at an initial pH of 5.0 or 9.0, respectively (Fig. 7b, c). At an improper pH, the results revealed a remarkable improvement of the lycopene yield with the help of the regulation circuits. The lycopene yield of the engineered strain BW-pUCASC increased by approximately 6.1-fold compared to the original strain BW-BIE at an initial pH 5.0 (*P* = 0.0061 < 0.01). The yield of BW-pUCBSC was nearly 4.0-fold higher than original strain at an initial pH 9.0 (*P* = 0.0073 < 0.01). This result suggested that the regulation circuits could significantly boost the pH resistance ability of the strains at an improper pH and improve the production of lycopene. When the initial pH was neutral, the lycopene yields of BW-pUCASC and BW-pUCBSC were 15.8% and 13.9% higher than original strain, respectively.

The lycopene production in BW-pUCATB with combined pH shooting circuit GPS was also analyzed. From the result, the lycopene yield of BW-pUCATB showed nearly 500% increase compared with the original strain at pH 5.0 (Fig. 7d) (*P* = 0.009 < 0.01). Similarly, at pH 6.0 and 6.5, the fermentation outputs of BW-pUCATB increased by 115.2% (*P* = 0.0072 < 0.01) and 40.66% (*P* = 0.0084 < 0.01) compared with BW-BIE, respectively. At pH values of 9.0, 8.0 and 7.5, the lycopene biosynthesis yield of BW-pUCATB showed 330.0% (*P* = 0.0069 < 0.01), 32.8% (*P* = 0.0075 < 0.01) and 53.0% (*P* = 0.0063 < 0.01) increases compared with the control, respectively. The pH alleviation ability of BW-pUCATB was slightly higher than that of the control strain BW-BIE, which further proved the function of GPS in pH control and production improvement. Therefore, an intelligent microbial cell factory might be able to be developed based on the GPS.

### Application of GPS in scale-up fermentation for increasing lycopene production

**Fig. 8:**
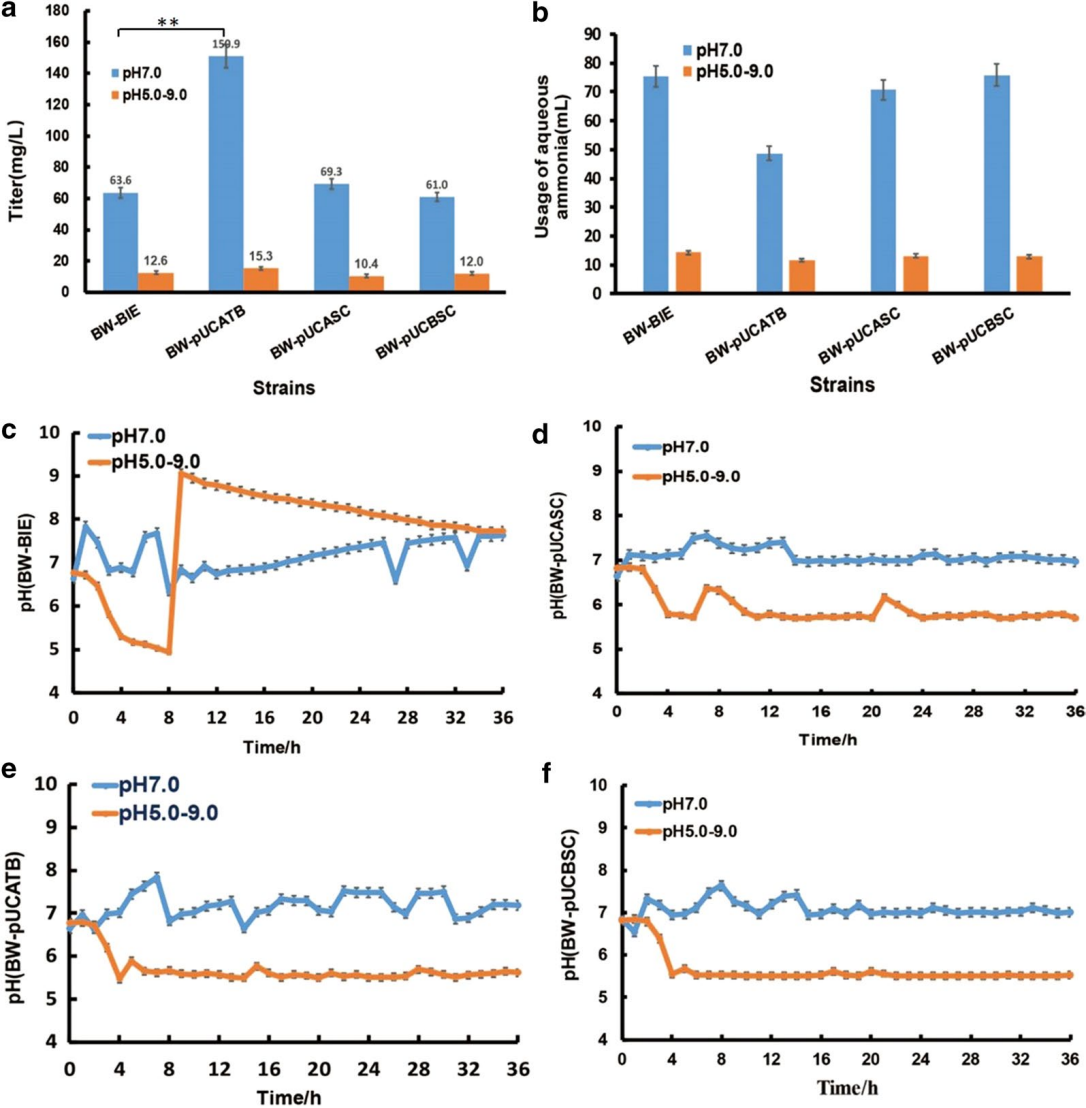
Lycopene fermentation of the engineered strains in batch fermenters with a stringent pH control manner (pH = 7) and a relaxed pH control manner (pH = 5 ~ 9), respectively. **a** lycopene yield at 24 h; **b** Usage of 20% aqueous ammonia after 36 h; **c–f** pH fluctuations for strain BW-BIE, BW-pUCASC, BW-pUCATB and BW-pUCBSC, respectively

The constructed GPS circuit could be furtherly applied to scale-up fermentation. Here, each engineered lycopene producing strain, BW-pUCASC, BW-pUCBSC and BW-pUCATB, was inoculated into a 5.0 L fermenter under stringent pH control mode (pH = 7.0) or relaxed pH control mode (pH = 5.0 ~ 9.0) (Fig. 8). For the stringent control manner, strain BW-pUCATB showed obvious higher lycopene concentration (increased by 137.3%) (*P* = 0.008 < 0.01) and less aqueous ammonia usage (decreased by 35.6%) compared to the control strain BW-BIE (Fig. 8a, b). Whereas, BW-pUCASC and BW-pUCBSC did not show significant improvement on lycopene production or ammonia usage. The dynamic variation of pH values revealed that pH stabilization has very tight correlation with lycopene production (Fig. 8c–f). BW-pUCATB showed the minimum pH fluctuation than any other strains, ensuring the highest lycopene production (150.9 mg/L) in this study. In addition, relaxed control mode for all the strains cannot lead to a high lycopene production, because the pH values deviate from 7.0 from the beginning. This result implied the great importance of precise pH control in maintain high production efficiency in fermentation process.

## Discussion

In microbial cultivation process, the medium is usually under the optimal pH at the beginning but becomes basic or acidic along with the production of various metabolites, which requires pH regulation for the better performance of microorganisms. The most common method to regulate pH is combining the probe detection and adding acid or alkali solutions into the cultures subsequently, which is costly and sometimes time-delay, restricting the industrial production. Nowadays, genetic circuits are usually designed for the purpose of regulation in synthetic biology. In this study, intelligent genetic circuits named GPS was constructed to realize self-regulation of pH. To achieve autonomous pH regulation, the pH-sensing promoters P-asr and P-atp2 reported before were utilized and tested. The gradually increased promoter activity of P-asr with pH decreasing suggested that the downstream functional gene would be expressed with the increase of H^+^, indicating that in this pH range, the more acidic of the environment, the stronger of the pH control ability of the acid regulation genetic circuit would be. The results showed that the activity of P-asr gradually decreased with increasing pH, roughly consistent with previous reports [22]. Meanwhile, the transcriptions of genes under P-asr are inhibited at neutral pH, avoiding too much burden for cell growth and production. In order to meet different pH control requirements, different mutants of P-atp2 were constructed in this study (Fig. 3). These diverse promoter mutants provide more candidates for different situations. For instance, the mutant-430 would be more helpful if accurate pH shooting is needed at a specific pH value. Here, wild-type promoter P-atp2 was employed because it can provide cells with a gradually increased pH homeostasis ability when the exterior pH rises. In future study, more promoters could be developed by mutation or mining to support diverse pH requirements.

In many cases, some bases and acids are produced along with the synthesis of products [10, 23]. For example, acidic environment appears frequently during some fermentation processes [24]. Therefore, different genetic circuits for pH self-regulation including single pH shooting circuits (ASC and BSC) and GPS were constructed and characterized. ASC and BSC could be employed in fermentation process with the culture becoming acid or alkali, respectively, while GPS was more general since both acid and alkali regulation could be conducted, broadening the applications of pH self-regulation in diverse situations. The results of the pH regulation ability test showed that GPS could adjust the external pH to an optimal value by themselves. The self-regulation of pH resulted from GPS was a novel strategy to improve the ability of microbial cell factories. Moreover, intelligent microbial production could be achieved in the near future.

Admittedly, there are still potential downsides of the pH self-regulation genetic circuits established in this study. The trade-offs between the production and pH homeostasis when using the pH-control genetic circuits developed in this study introduced uncertainty to the production. For example, the regulating compounds generated from the acid-production gene and base-production gene may introduce undesired by-products in the fermentation, increasing the difficulties in downstream separation. The acid-production gene or basic production gene may compete for substrates with the cell growth or biosynthesis pathway, which may reduce the theoretical yields and final titers of target products. However, the goal of designing such circuits is to prove the feasibility of autonomous pH regulation by microorganisms, so only the proof-of-concept genetic circuits were built and tested in this study. Many engineering efforts, such as pH-sensitive promoter engineering, acid- or base-production gene mining, and utilizing different substances to minimize the cell burden and competition with biosynthesis pathway, could be carried out to improve the future performance and applicability of these circuits.

Lycopene is a type of natural pigment and active compound that is commonly utilized in foods, cosmetics, nutritional supplements, and pharmaceuticals due to its strong antioxidation ability [25, 26]. The color of lycopene makes it convenient to detect the concentration. Hence, lycopene was employed as an example to verify the function of GPS in microbial cell factories. Fermentation results proved that lycopene production remained at a high level at an improper pH from 5.0 to pH 9.0 when GPS was used, which greatly expanded the pH range that the strain could maintain high efficiency compared with the wild-type strain. Under “stringent” condition, the GPS system showed significant advantage in producing lycopene compared with other strain. Under “relaxed” condition, the pUCATB strain also performed better than the other strains as Fig. 8a showed. But the superiority was not quite significant in scale-up fermentation because the pH changed dramatically during the first 12 h and lots of acid or alkali were needed to adjust the pH (Fig. 8c). Taking GPS system as the sole regulation approach was insufficient in the scale-up fermentation since the GPS system constructed in this study was still an original version without engineering. As discussed before, the GPS could be improved in the future by engineering the promoters or the corresponding acid- or base-production genes to achieve the complete self-regulation in scale-up fermentation. Moreover, the production cost could be greatly reduced for the improvement of lycopene concentration and decrease of acid or alkali solutions usage.

Overall, the GPS discussed in this study provides a new strategy in the pH regulation and could be applied in many areas such as the research on diverse self-regulation in the construction of microbial cell factories, the improvement of robustness for engineered strains, and the reducing of production cost. More importantly, the concept of pH self-regulation could be the foundation of constructing intelligent microbial cell factories.

## Conclusions

In this study, we proposed a novel concept to achieve self-responsive regulation of the extracellular pH through intelligent genetic circuits. Two promotors, P-asr and P-atp2, and three genes including *ldhA*, *glsA* and *gadA*, were mined and employed to construct pH self-regulating circuits, i.e. GPS. In order to broaden the applications of pH self-regulation in diverse situations, single pH shooting circuits, ASC and BSC, were also constructed to meet different requirements. The results showed that pH self-regulating circuits including GPS, BSC and ASC could help the cells to adjust the external pH to an optimal value by themselves. Therefore, cell growth was much better in the pH self-regulating strains than the wild type under non-neutral medium. The pH self-regulating circuits were also applied to the microbial cell factories to produce lycopene in this study. The yield of lycopene was improved after introducing GPS under pH ranging from 5.0 to 9.0. In fermenter experiments, more than 100% increase of lycopene concentration was achieved in the engineered *E. coli* BW-pUCATB compared with the control strain. In summary, GPS circuits show the high potential in enhancing cell production with boosted pH homeostasis and in reducing energy consumption and emission. Our strategy of GPS provides new insight into solving the pH regulation and will lay the foundations for intelligent microbial cell factories.

## Methods

### Strains, plasmids and media

The strains and plasmids used in this study were listed in Table 1. LB medium (5 g/L yeast extract, 10 g/L tryptone and 10 g/L NaCl) was used for strain cultivation. For the lycopene production strains, culture medium contained (g/L): 10 tryptone, 5 yeast extract, 20 glycerol, 12.8 Na_2_HPO_4_∙7H_2_O, 0.022 CaCl_2_, 3 KH_2_PO_4_, 0.5 NaCl, 0.48 MgSO_4_, 1 NH_4_Cl and 10 (NH_4_)_2_SO_4_.

**Table 1:** Strains and plasmids used in this study

Strains	Relevant genotype	Reference or source
*E. coli* BM Top 10	F^–^ *mcrA Δ(mrr-hsdRMS-mcrBC)* φ80*lacZ*Δ*M15* Δ*lacX74 nupG recA1 araD139 Δ(ara-leu)7697 galE15 galK16 rpsL(*Str^R^*) endA1* λ^−^	Purchased from biomed
*E. coli* BL21(DE3)	*E. coli* str. B F^–^ *ompT gal dcm lon hsdS*_*B*_(*r*_*B*_^–^*m*_*B*_^–^) λ(DE3) [*lacI lacUV5*-*T7p07 ind1 sam7 nin5*] [*malB*^+^]_K-12_(λ^S^)	Purchased from biomed
*E. coli* BW-BIE	The strains for lycopene biosynthesis	[27]
BM-pSB1C3	*E. coli* BM Top10 carrying the plasmid pSB1C3	This study
BM-pSBASC	*E. coli* BM Top10 carrying the plasmid pSB-ASC	This study
BM-pSBBSC	*E. coli* BM Top10 carrying the plasmid pSB-BSC	This study
BL-pET28a	*E. coli* BL21(DE3) carrying the plasmid pET28a	This study
BL-pETglsA	*E. coli* BL21(DE3) carrying the plasmid pET-glsA	This study
BL-pETgadA	*E. coli* BL21(DE3) carrying the plasmid pET-gadA	This study
BW-pUCASC	*E. coli* BW-BIE carrying the plasmid pUC-ASC	This study
BW-pUCBSC	*E. coli* BW-BIE carrying the plasmid pUC-BSC	This study
BW-pUCATB	*E. coli* BW-BIE carrying the plasmid pUC-ATB	This study
Plasmids		
pSB1C3	The wildly used backbone for gene cloning in synthetic biology	iGEM Registry
pUC19	Cloning vector, Amp^r^	Purchased from BioMed
pET28a	Expression vector, kan^r^	Lab Preserved
pSB-ASC	pSB1C3 carrying the acid regulation circuit (P-asr + glsA)	This study
pSB-BSC	pSB1C3 carrying the base regulation circuit (P-apt2 + ldhA)	This study
pETglsA	pET28a inserted by *glsA* gene in the multiple cloning sites	This study
pETgadA	pET28a inserted by *gadA* gene in the multiple cloning sites	This study
pUCASC	pUC19 carrying the acid shooting circuit (P-asr + glsA)	This study
pUCBSC	pUC19 carrying the base shooting circuit (P-atp2 + ldhA)	This study
pUCATB	pUC19 carrying the genetic pH shooting circuits with a terminator between the two circuits ASC and BSC	This study

### DNA manipulation

The plasmid pSB1C3 was from the iGEM Registry and was transformed into *E. coli str. K-12* BM Top10. The base-induced promoter P-atp2 was amplified from the genome of *C. glutamicum* through PCR*.* The acid-induced promoter P-asr and the three genes *ldhA, gadA* and *glsA* were amplified from the genome of *E. coli str. K-12* through PCR amplification. The *lacZα* gene was linked downstream of each promoter (including RBS) by overlap-extension PCR (OE-PCR). Those two gene circuits were both constructed on the plasmids pSB1C3 and pUC19 and then transformed into *E. coli* BM Top10. To construct the single pH shooting circuits, OE-PCR was used to combine the promoters and the corresponding functional gene RBS B0034. The PCR products were then purified and cloned into the vector pSB1C3, using *Xba*I and *Pst*I, resulting in pSB-ASC and pSB-BSC. These two circuits were ligated by OE-PCR to construct the GPS circuit in pUC19.

### Promoter activity assay

The *lacZα* gene was chosen as the reporter gene in this study to determine the activity of P-asr and P-atp2 at different pH values. The pH gradient was designed at pHs 4.0, 5.0, 6.0 and 7.0 for P-asr and pHs 6.0, 7.0, 8.0, 9.0 and 9.7 for P-atp2 (when the pH was higher than 10.0, the bacteria aggregated together and lysed). The methods used for the determination of enzymatic activity were the same for the two gene circuits and for both the control group and experimental group, which is described as follows. Cultures were incubated in LB medium at 37 °C for 8–10 h and then diluted into fresh LB medium with a ratio of 4%. When the OD_600_ value of the bacteria reached approximately 0.6, sterile H_2_SO_4_ or NaOH was added to the medium to adjust the pH, and the pH was detected by a pH test pen. New cultures were grown for another 12 h, and the final OD_600_ values were recorded for each culture. 1.0 mL sample was taken from each culture and centrifuged at 12,000 rpm for 1 min, respectively. The supernatant was discarded. 1 mL of PBS (pH 7.4) was then added to each sample, followed by centrifugation and removal of the supernatant. This process was repeated three times. Then, 1 mL PBS (pH 7.4) was added and mixed with sediment, and the samples were placed on ice. Ultrasonic waves were used to break the cells of each strain, with 8 min for each sample. All of the processed samples were centrifuged at 4 °C, 7500 rpm for 10 min. The supernatant of each sample was transferred to a 96-well plate in triplicate to decrease accidental errors, with each well containing 150 μL supernatant; 40 μL 4 g/l ortho-Nitrophenyl-β-galactoside (ONPG) was then added. The plate was placed in an incubator at 37 °C, and the initial time was recorded. The end time was the time when the reacting solution in some of the wells turned yellow. 60 μL of the Na_2_CO_3_ solution (1 mol/L) was added to stop the reaction. OD_420_ and OD_550_ were then recorded via a microplate reader (BioTek, VT, US). The activity of the enzyme was calculated based on the formula below:

$$ Activity_{{enzyme}}  = 1000 \times \frac{{A_{{420}} \; - \;(1.75 \times A_{{550}} )}}{{t \times 0.1 \times A_{{600}} }}  $$

where $t = t_{initial} - t_{end},$ A represents absorbance.

### EP-PCR

Stratagene's Genemorph.II Random MUtagenesis kit was used to perform error-prone PCR amplification of gene sequences. The reaction system of EP-PCR was shown in Additional file 1: Table S1, and the reaction conditions of EP-PCR was shown in Additional file 1: Table S2. The primers used for EP-PCR were listed in Additional file 1: Table S3. The standard protocol for Genemorph II kit was followed for the EP-PCR.

### Verification of pH control ability

Transformants of BM Top10 carrying the pSB-ASC and pSB-BSC were cultivated in 40 mL LB medium for 12 h and then diluted into new medium at a 2% ratio. When the bacteria grew to an approximate OD_600_ of 0.6, sterile H_2_SO_4_ or NaOH was added to the medium to adjust the pH to different values. The acid shooting circuit (ASC) was tested from pH 4.0 to 7.0 and grown at 37 °C, while the base shooting circuit (BSC) was tested from pH 7.0 to 9.7 and grown at 37 °C.

### Fermentation of the lycopene production strains

BW-pUCATB, BW-pUCASC and BW-pUCBSC (the strains carrying the regulation circuits) were cultivated in 20 mL LB medium for 8–10 h and then diluted into fresh lycopene fermentation medium at a ratio of 5%. Sterile H_2_SO_4_ or NaOH was added to the medium to reach the designated pH values. The control group was the original strain without a regulation circuit. All groups were cultivated for 24 h.

### Scale-up fermentation

Scale-up fermentation was performed in a 5 L fermenter with a working volume of 3 L. The fermentation medium includes 1.5% peptone, 1.2% yeast extract, 2% glucose, 0.3% NaH_2_P0_4_∙2H_2_0, 0.7% K_2_HP0_4_∙3H_2_0, 0.25% NaCl, 0.5% tween80. The fermentation temperature was set to 37 °C. 20% NH_3_·H_2_O and 1 M HCl were used to control the pH within the set range by the PID controller. The rotating speed was set to 500 rpm, and air flow rate was 3 L/h. After 24 h of fermentation, the lycopene titer was measured for each sample.

### Sampling and extraction of lycopene

A 1.0 mL sample of each culture was extracted from both groups after 24 h of fermentation. Samples were centrifuged at 12,000 rpm for 1 min, and the supernatant was discarded. Then, each sample was resuspended in 1.0 mL PBS (pH = 7.4), followed by centrifugation and the removal of the supernatant. 800 µL acetone was added to each sample for the extraction of lycopene and cell lysis. Samples were heated in a water bath at 50 °C for 15 min and then centrifuged at 12,000 rpm for 1 min. The supernatant was kept for measurement at OD_472_. A standard curve of lycopene dissolved in acetone was obtained with commercial lycopene.

## Supplementary information

**Additional file 1: Table S1.** Reaction system of EP-PCR.** Table S2.** Reaction conditions of EP-PCR.** Table S3.** Sequence of primers used for EP-PCR. Figure S1. Growth curve of the scale-up fermentations.

## Data Availability

The datasets used and analyzed during the current study are available from the corresponding author on reasonable request.

## Abbreviations

GPS: Genetic pH shooting
ACS: Acid shooting circuit
BSC: Base shooting circuit
