# Supplementary material for: Intelligent microbial cell factory with genetic pH shooting (GPS) for cell self-responsive base/acid regulation
Source: Microb Cell Fact. 2020 Nov 2;19:202. doi: 10.1186/s12934-020-01457-3 (PMC7607686; doi:10.1186/s12934-020-01457-3)
Supplement: Supplementary file 1 — Additional file 1: Table S1. Reaction system of EP-PCR. Table S2. Reaction conditions of EP-PCR. Table S3. Sequence of primers used for EP-PCR. Figure S1. Growth curve of the scale-up fermentations. [file 12934_2020_1457_MOESM1_ESM.docx]

Additional file 1

## Table S1ReactionsystemofEP-PCR

| Reaction system | Amount |
| --- | --- |
| 10×Mutazyme II reaction buffer | 5 μL |
| 40 mM dNTP mix | 1 μL |
| Forward prime | 0.5 μL |
| Reverse prime | 0.5 μL |
| Mutazyme II DNA polymerase | 1 μL |
| template | 1 μL |
| ddH_2_O | 41 μL |

## Table S2 Reaction conditions of EP-PCR

| Temperature | Time | Cycle |
| --- | --- | --- |
| 95℃ | 2 min | 1 cycle |
| 95℃ | 0.5 min |  |
| 95℃ | 0.5 min | 30 cycles |
| 95℃ | 2 min |  |
| 95℃ | 10 min | 1 cycle |

Table S3 Sequence of primers used for EP-PCR

| Primer name | Primer sequence | Restriction site |
| --- | --- | --- |
| EP-P-atp2-F | AAGCTTAGTGTCCGTGCTGGGAAACT | *Hind*III |
| EP-P-atp2-R | GGATCCCGGACTCCGTCGCACATCAG | *Bam*HI |

Figure S1 Growth curve of the scale-up fermentations


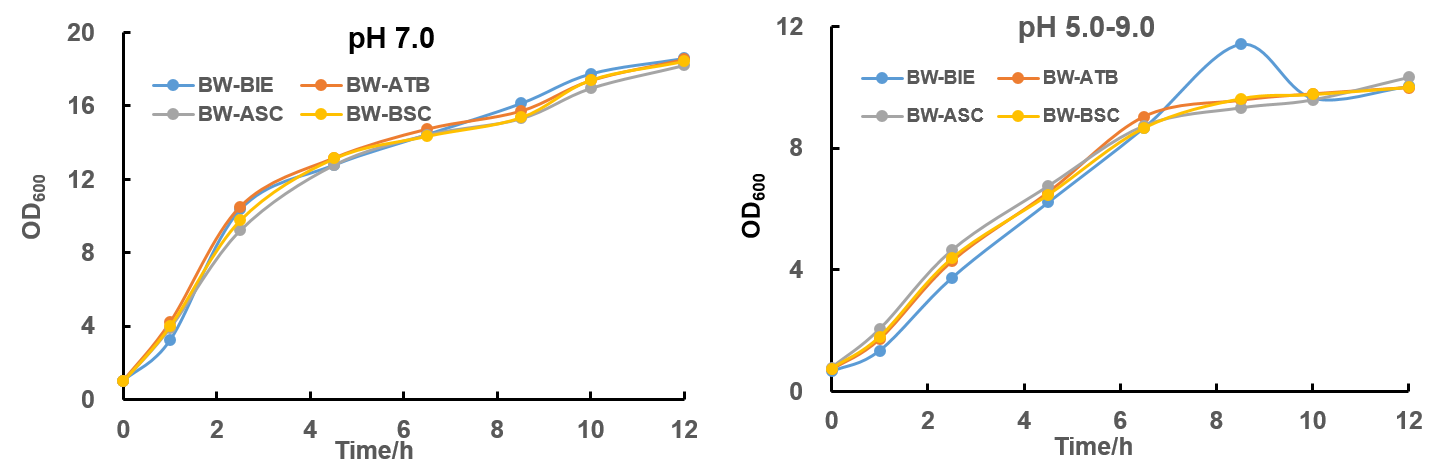


DNA sequence of P-atp2

AGTGTCCGTGCTGGGAAACTGGCGGGGAACTTTTAGAGATAACCCTCCGAATTGCTGGCAAATTTCTGATGAAATTTCTCCGCGAAGCCCACATGAACTACCCCCGTTTACCCTCAAAATAAGCCCTGTGACACACATAACACCCCCTAATCGTACCCGCTCACACGCTATTTTCGAGGTGTGGTTCGCCTTCGGAAACGAATGCCCCCGCCCCACTTGGATAAAAGACGAATTCACCTGTTAGTCTATAACGCGGGTTGAACCGAGAAACCCCTCAAGGCAGCAGACAATAGCCGCAAGGGGTTTTGCGGAGCACGTCCCCTGTGATCGTTGCGCTGATGTGCGACGGAGTCCGG

DNA sequence of P-atp2 Mutant-140

AGTGTCCGTGCTGGGAAACTGGCGGGGAACTTTTAGAGATAACCCTCCGAATTGCTGGCAAATTTCTGATGAAATTTCTCCGCGAAGCCCACATGAACTACCCCCGTTTACCCTCAAAATAAGCCCTGTGACACACATAACACCCCCTAATCGTACCCGCTCACACGCTATTTTCGAGGTGTGGTTCGCCTTCGGAAACGAATGCCCCCGCCCCACTTGGATAAAAGACGTTAACACCTGTTAGTCTATAACGCGGGTTGAACCGAGAAACCCCTCAAGGCAGCAGACAATAGCCGCAAGGGGTTTTGCGGAGCACGTCCCCTGTGATCGTTGCGCTGATGTGCGACGGAGTCCG

DNA sequence of P-atp2 Mutant-154

AGTGTCCGTGCTGGGAAACTGGCGGGGAACTTTTAGAGATAACCCTCCGAATTGCTGGCAAATTTCTGATGAAATTTCTCCGCGAAGCCCACATGAACTACCCCCGTTTACCCTCAAAATAAGCCCTGTGACACACATAACACCCCCTAATCGTACCCGCTCACACGCTATTTTCGAGGTGTGGTTCGCCTTCGGAAACGAATGCCCCCGCCCCACTTGGATAAAAGACGTTAACACCTGTTAGTCTATAACGCGGGTTGAACCGAGAAACCCCTCAAGGCAGCAGACAATAGCCGCAAGGGGTTTTGCGGAGCACTTCCCCTGTGATCGTTGCGCTGATGTGCGACGGAGTCCG

DNA sequence of P-atp2 Mutant-202

AGTGTCCGTGCTGGGAAACTGGCGGGGAACTTTTAGAGATAACCCTCCGAATTGCTGGCAAATTTCTGATGAAATTTCTCCGCGAAGCCCACATGAACTACCCCCGTTTACCCTCAAAATAAGCCCTGTGACACACATAACACCCCCTAATCGTACCCGCTCACACGCTATTTTCGAGGTGTGGTTCGCCTTCGGAAACGAATGCCCCCGCCCCACTTGGATAAAAGACGTTAACACCTGTTAGTCTATAACGCGGGTTGAACCGAGAAACCCCTCAAGGCAGCAGACAATAGCCGCAAGGGGTTTTGCGGAGCACGTCCCCTGTGATCGTCGCGCTGATGTGCGACGGAGTCCG

DNA sequence of P-atp2 Mutant-226

AGTGTCCGTGCTGGGAAACTGGCGGGGAACTTTTAGAGATAACCCTCCGAATTGCTGGCAAATTTCTGATGAAATTTCTCCGCGAAGCCCACATGAACTACCCCCGTTTACCCTCAAAATAAGCCCTGTGACACACATAACACCCCCTAATCGTACCCGCTCACACGCTATTTTCGAGGTGTGGTTCGCCTTCGGAAACGAATGCCCCCGCCCCACTTGGATAAAAGACGAATTCACCTGTTAGTCTATAACGCGGGTTGAACCGAGAAACCCCTCAAGGCAGCAGACAATAGCCGCAAGGGGTTTTGCGGAGCACGTCCCCTGTGATCGTTGCGCTGATGTGCGACGGAGCCCG

DNA sequence of P-atp2 Mutant-317

AGTGTCCGTGCTGGGAAACTGGCGGGGAACTTTTAGAGATAACCCTCCGAATTGCTGGCAAATTTCTGATGAAATTTCTCCGCGAAGCCCACATGAACTACCCCCGTTTACCCTCAAAATAAGCCCTGTGACACACATAACACCCCCTAATCGTACCCGCTCACACGCTATTTTCGAGGTGTGGTTCGCCTTCGGAAACGAATGCCCCCGCCCCACTTGGATAAAAGACGAATTCACCTGTTAGTCTATAACGCGGGTTGAACCGAGAAACCCCTCAAGGCAGCAGACAATAGCCGCAAGGGGTTTTGCGGAGCACGTCGCCTGTGATCGTTGCGCTGATGTGCGACGGAGTCCG

DNA sequence of P-atp2 Mutant-399

AGTGTCCGTGCTGGGAAACTGGCGGGGAACTTTTAGAGATAACCCTCCGAATTGCTGGCAAATTTCTGATGAAATTTCTCCGCGAAGCCCACATGAACTACCCCCGTTTACCCTCAAAATAAGCCCTGTGACACACATAACACCGCCTAATCGTACCCGCTCACACGCTATTTTCGAGGTGTGGTTCGCCTTCGGAAACGAATGCCCCCGCCCCACTTGGATAAAAGACGAATTCACCTGTTAGTCTATAACGCGGGTTGAACCGAGAAACCCCTCAAGGCAGCAGACAATAGCCGCAAGGGGTTTTGCGGAGCACGTCCCCTGTGATCGTTGCGCTGATGTGCGACGGAGTCCG

DNA sequence of P-atp2 Mutant-430

AGTGTCCGTGCTGGGAAACTGGCGGGGAACTTTTAGAGATAACCCTCCGAATTGCTGGCAAATTTCTGATGAAATTTCTCCGCGAAGCCCACATGAACTACCCCCGTTTACCCTCAAAATAAGCCCTGTGTGACACACATAACACCCCCTAATCGTACCCGCTCACACGCTATTTTCGAGGTGTGGTTCGCCTTCGGAAACGAATGCCCCCGCCCCACTTGGATAAAAGACGTTAACACCTGTTAGTCTATAACGCGGGTTGAACCGAGAAACCCCTCAAGGCAGCAGACAATAGCCGCAAGGGGTTTTGCGGAGCACGTCCCCTGTGATCGTTGCGCTGATGTGCGACGGAGTCCG

DNA sequence of P-atp2 Mutant-705

AGTGTCCGTGCTGGGAAACTGGCGGGGAACTTTTAGAGATAACCCTCCGAATTGCTGGCAAATTTCTGATGAAATTTCTCCGCGAAGCCCACATGAACTACCCCCGTTTACCCTCAAAATAAGCCCTGTGACACACATAACACCCCCTAATCGTACCCGCTCACACGCTATTTTCGAGGTGTGGTTCGCCTTCGGAAACGAATGCCCCCGCCCCACTTGGATAAAAGACGTTAACACCTGTTAGTCTATAACGCGGGTTGAACCGAGAAACCCCTCAAGGCAGCAGACAATAGCCGCAGGGGTTTTGCGGAGCACGTCCCCTGTGATCGTTGCGCTGATGTGCGACGGAGTCCG

DNA sequence of P-atp2 Mutant-706

AGTGTCCGTGCTGGGAAACTGGCGGGGAACTTTTAGAGATAACCCTCCGAATTGCTGGCAAATTTCTGATGAAATTTCTCCGCGAAGCCCACATGAACTACCCCCGTTTACCCTCAAAATAAGCCCTGTGACACACATAACACCCCCTAATCGTACCCGCTCACACGCTATTTTCGAGGTGTGGTTCGCCTTCGGAAACGAATGCCCCCGCCCCACTTGGATAAAAGACGAATTCACCTGTTAGTCTATAACGCGGGTTGAACCGAGAAACCCCTCAAGGCAGCAGACAATAGCCGCAAGGGGTTATGCGGAGCACGTCCCCTGTGATCGTTGCGCTGATGTGCGACGGAGTCCG

DNA sequence of P-asr

CGATCAAGACTACTATTATTGGTAGCTAAATTTCCCTTAAGTCACAATACGTTATTATCAACGCTGTAATTTATTCAGCGTTTGTACATATCGTTACACGCTGAAACCAACCACTCACGGAAGTCTGCCATTCCCAGGGATATAGTTATTTCAACGGCCCCGCAGTGGGGTTAAATGA

DNA sequence and gene accession number of *ldh*A

Gene accession number: 946315

ATGAAACTCGCCGTTTATAGCACAAAACAGTACGACAAGAAGTACCTGCAACAGGTGAACGAGTCCTTTGGCTTTGAGCTGGAATTTTTTGACTTTCTGCTGACGGAAAAAACCGCTAAAACTGCCAATGGCTGCGAAGCGGTATGTATTTTCGTAAACGATGACGGCAGCCGCCCGGTGCTGGAAGAGCTGAAAAAGCACGGCGTTAAATATATCGCCCTGCGCTGTGCCGGTTTCAATAACGTCGACCTTGACGCGGCAAAAGAACTGGGGCTGAAAGTAGTCCGTGTTCCAGCCTATGATCCAGAGGCCGTTGCTGAACACGCCATCGGTATGATGATGACGCTGAACCGCCGTATTCACCGCGCGTATCAGCGTACCCGTGATGCTAACTTCTCTCTGGAAGGTCTGACCGGCTTTACTATGTATGGCAAAACGGCAGGCGTTATCGGTACCGGTAAAATCGGTGTGGCGATGCTGCGCATTCTGAAAGGTTTTGGTATGCGTCTGCTGGCGTTCGATCCGTATCCAAGTGCAGCGGCGCTGGAACTCGGTGTGGAGTATGTCGATCTGCCAACCCTGTTCTCTGAATCAGACGTTATCTCTCTGCACTGCCCGCTGACACCGGAAAACTATCATCTGTTGAACGAAGCCGCCTTCGAACAGATGAAAAATGGCGTGATGATCGTCAATACCAGTCGCGGTGCATTGATTGATTCTCAGGCAGCAATTGAAGCGCTGAAAAATCAGAAAATTGGTTCGTTGGGTATGGACGTGTATGAGAACGAACGCGATCTATTCTTTGAAGATAAATCCAACGACGTGATCCAGGATGACGTATTCCGTCGCCTGTCTGCCTGCCACAACGTGCTGTTTACCGGGCACCAGGCATTCCTGACAGCAGAAGCTCTGACCAGTATTTCTCAGACTACGCTGCAAAACTTAAGCAATCTGGAAAAAGGCGAAACCTGCCCGAACGAACTGGTTTAA

Sequence and gene accession number of *gls*A

Gene accession number: 946187

ATGTTAGATGCAAACAAATTACAGCAGGCAGTGGATCAGGCTTACACCCAATTTCACTCACTTAACGGCGGACAAAATGCCGATTACATTCCCTTTCTGGCGAATGTACCAGGTCAACTGGCGGCAGTGGCTATCGTGACCTGCGATGGCAACGTCTATAGTGCGGGTGACAGTGATTACCGCTTTGCACTGGAATCCATCTCGAAAGTCTGTACGTTAGCCCTTGCGTTAGAAGATGTCGGCCCGCAGGCGGTACAGGACAAAATTGGCGCTGACCCGACCGGATTGCCCTTTAACTCAGTTATCGCCTTAGAGTTGCATGGCGGCAAACCGCTTTCGCCACTGGTAAATGCTGGCGCTATTGCCACCACCAGCCTGATTAACGCTGAAAATGTTGAACAACGCTGGCAGCGAATTTTA

CATATCCAACAGCAACTGGCTGGCGAGCAGGTAGCGCTCTCTGACGAAGTCAACCAGTCGGAACAAACAACCAACTTCCATAACCGGGCCATAGCCTGGCTGCTGTACTCCGCCGGATATCTCTATTGTGATGCAATGGAAGCCTGTGACGTGTATACCCGTCAGTGCTCCACGCTCCTCAATACTATTGAACTGGCAACGCTTGGCGCGACGCTGGCGGCAGGTGGTGTGAATCCGTTGACGCATAAACGCGTTCTTCAGGCCGACAACGTGCCGTACATTCTGGCCGAAATGATGATGGAAGGGCTGTATGGTCGCTCCGGTGACTGGGCGTATCGTGTTGGTTTACCGGGCAAAAGCGGTGTAGGTGGCGGTATTCTGGCGGTCGTCCCTGGAGTGATGGGAATTGCCGCGTTCTCA

CCACCGCTGGACGAAGATGGCAACAGTGTTCGCGGTCAAAAAATGGTGGCATCGGTCGCTAAGCAACTCGGCTATAACGTGTTTAAGGGCTGA

Sequence and gene accession number of *gad*A

Gene accession number: 948027

ATGGACCAGAAGCTGTTAACGGATTTCCGCTCAGAACTACTCGATTCACGTTTTGGCGCAAAGGCCATTTCTACTATCGCGGAGTCAAAACGATTTCCGCTGCACGAAATGCGCGATGATGTCGCATTTCAGATTATCAATGATGAATTATATCTTGATGGCAACGCTCGTCAGAACCTGGCCACTTTCTGCCAGACCTGGGACGACGAAAACGTCCATAAATTGATGGATTTGTCGATCAATAAAAACTGGATCGACAAAGAAGAATATCCGCAATCCGCAGCCATCGACCTGCGTTGCGTAAATATGGTTGCCGATCTGTGGCATGCGCCTGCGCCGAAAAATGGTCAGGCCGTTGGCACCAACACCATTGGTTCTTCCGAGGCCTGTATGCTCGGCGGGATGGCGATGAAATGGCGTTGGCGCAAGCGTATGGAAGCTGCAGGCAAACCAACGGATAAACCAAACCTGGTGTGCGGTCCGGTACAAATCTGCTGGCATAAATTCGCCCGCTACTGGGATGTGGAGCTGCGTGAGATCCCTATGCGCCCCGGTCAGTTGTTTATGGACCCGAAACGCATGATTGAAGCCTGTGACGAAAACACCATCGGCGTGGTGCCGACTTTCGGCGTGACCTACACCGGTAACTATGAGTTCCCACAACCGCTGCACGATGCGCTGGATAAATTCCAGGCCGACACCGGTATCGACATCGACATGCACATCGACGCTGCCAGCGGTGGCTTCCTGGCACCGTTCGTCGCCCCGGATATCGTCTGGGACTTCCGCCTGCCGCGTGTGAAATCGATCAGTGCTTCAGGCCATAAATTCGGTCTGGCTCCGCTGGGCTGCGGCTGGGTTATCTGGCGTGACGAAGAAGCGCTGCCGCAGGAACTGGTGTTCAACGTTGACTACCTGGGTGGTCAAATTGGTACTTTTGCCATCAACTTCTCCCGCCCGGCGGGTCAGGTAATTGCACAGTACTATGAATTCCTGCGCCTCGGTCGTGAAGGCTATACCAAAGTACAGAACGCCTCTTACCAGGTTGCCGCTTATCTGGCGGATGAAATCGCCAAACTGGGGCCGTATGAGTTCATCTGTACGGGTCGCCCGGACGAAGGCATCCCGGCGGTTTGCTTCAAACTGAAAGATGGTGAAGATCCGGGATACACCCTGTACGACCTCTCTGAACGTCTGCGTCTGCGCGGCTGGCAGGTTCCGGCCTTCACTCTCGGCGGTGAAGCCACCGACATCGTGGTGATGCGCATTATGTGTCGTCGCGGCTTCGAAATGGACTTTGCTGAACTGTTGCTGGAAGACTACAAAGCCTCCCTGAAATATCTCAGCGATCACCCGAAACTGCAGGGTATTGCCCAGCAGAACAGCTTTAAACACACCTGA
